# COVID-19 pandemic dynamics in India, the SARS-CoV-2 Delta variant and implications for vaccination

**DOI:** 10.1098/rsif.2021.0900

**Published:** 2022-06-06

**Authors:** Wan Yang, Jeffrey Shaman

**Affiliations:** ^1^ Department of Epidemiology, Columbia University, New York, NY, USA; ^2^ Department of Environmental Health Sciences, Mailman School of Public Health, Columbia University, New York, NY, USA

**Keywords:** COVID-19, Delta SARS-CoV-2 variant, India, vaccine effectiveness, boosting, prior infection

## Abstract

The Delta variant is a major SARS-CoV-2 variant of concern first identified in India. To better understand COVID-19 pandemic dynamics and Delta, we use multiple datasets and model-inference to reconstruct COVID-19 pandemic dynamics in India during March 2020–June 2021. We further use the large discrepancy in one- and two-dose vaccination coverage in India (53% versus 23% by end of October 2021) to examine the impact of vaccination and whether prior non-Delta infection can boost vaccine effectiveness (VE). We estimate that Delta escaped immunity in 34.6% (95% CI: 0–64.2%) of individuals with prior wild-type infection and was 57.0% (95% CI: 37.9–75.6%) more infectious than wild-type SARS-CoV-2. Models assuming higher VE among non-Delta infection recoverees, particularly after the first dose, generated more accurate predictions than those assuming no such increases (best-performing VE setting: 90/95% versus 30/67% baseline for the first/second dose). Counterfactual modelling indicates that high vaccination coverage for first vaccine dose in India combined with the boosting of VE among recoverees averted around 60% of infections during July–mid-October 2021. These findings provide support to prioritizing first-dose vaccination in regions with high underlying infection rates, given continued vaccine shortages and new variant emergence.

## 1. Introduction

The Delta variant (PANGO lineage: B.1.617.2) is a major SARS-CoV-2 variant of concern (VOC) [1–4] that spread to at least 200 countries and territories (Global Initiative on Sharing All Influenza Data (GISAID) [5], as of 28 March 2022). Several lines of evidence have indicated that Delta is able to evade immunity from prior infection by pre-existing variants; these include reduced neutralizing ability of convalescent sera and vaccinee sera against Delta [6–9], reduced vaccine effectiveness (VE) against infection [10–13] and reduced VE against symptomatic disease after first-dose vaccine (but only slight reduction for full vaccination) [14–16]. In addition, studies have found a higher secondary attack rate, growth rate or reproduction number for Delta than prior variants including Alpha (range of the mean estimates: 60–120%) [2,17–21], In particular, Dhar *et al*. [22], fitting a model to mortality data in Delhi, India, estimated a 1.3-fold to 1.7-fold (50% CI) increase in transmissibility and 10–50% (50% CI) immune evasion for Delta; however, the authors noted large uncertainty in their estimates [22]. Further, factors such as host behavioural changes and seasonal modulation of risk due to changes in environmental conditions are difficult to account for and could confound these estimates. As a result, estimates of prior immunity evasion and relative transmissibility for Delta and the contributions of these properties to the rapid spread of this variant remain uncertain.

India, where Delta was first identified, experienced an intense pandemic wave in late March 2021. However, unlike many places seeing a prolonged Delta pandemic wave, the Delta wave in India only lasted three months and declined rapidly after peaking mid-May. Cases remained low during June–October 2021 (the time of this study). A high infection rate after the Delta wave has been cited as a reason for this dramatic epidemic decline, as vaccination coverage was low at the time (4.2% fully vaccinated at the end of June 2021). However, given an estimated basic reproduction number (*R*_0_) of 6–7 [20], roughly 83–86% (1–1/*R*_0_) of the population would need immunity for the Delta epidemic to subside. Assuming 10–50% immunity escape [22] and a 25–35% infection rate prior to the Delta wave [23], this implies that 53–73% of India's 1.4 billion people would have been infected by Delta within the span of three months, despite a national lockdown at the time.

To better understand COVID-19 pandemic dynamics in India and the epidemiological characteristics of Delta, here we use a model-inference method recently developed for SARS-CoV-2 VOCs. The model-inference method incorporates epidemiological, population mobility and weather data to model SARS-CoV-2 transmission dynamics, while accounting for case under-ascertainment, impacts of non-pharmaceutical interventions (NPIs) and vaccination, infection seasonality and new variants [24]. Applying this method, we have jointly estimated the immune escape potential and change in transmissibility for Alpha, Beta and Gamma, separately, using data from countries where these three VOCs were first reported [24]. In addition, several laboratory studies have reported stronger vaccine-induced immune responses among recovered vaccinees than naive vaccinees, suggesting potential boosting of pre-existing immunity [25–28]. In India, while only 23% of the population have received two vaccine doses, 53% have received their first vaccine dose, as of the end of October 2021. This large discrepancy in one- and two-dose coverage, combined with a likely high population infection rate, offers an opportunity to examine the boosting effect of prior non-Delta infection on vaccine-induced immunity at the population level. Therefore, in this study, we first reconstruct the pandemic dynamics in India during March 2020–June 2021 and estimate key epidemiological characteristics of Delta. We then further use our model estimates to retrospectively predict cases and deaths during July–October 2021, under various vaccination and VE scenarios, and compare these simulations to observations in order to estimate the impact of vaccination and VE for those with prior non-Delta infection.

## 2. Results

### 2.1. The first COVID-19 pandemic wave in India, March 2020–January 2021

**Figure 1.**
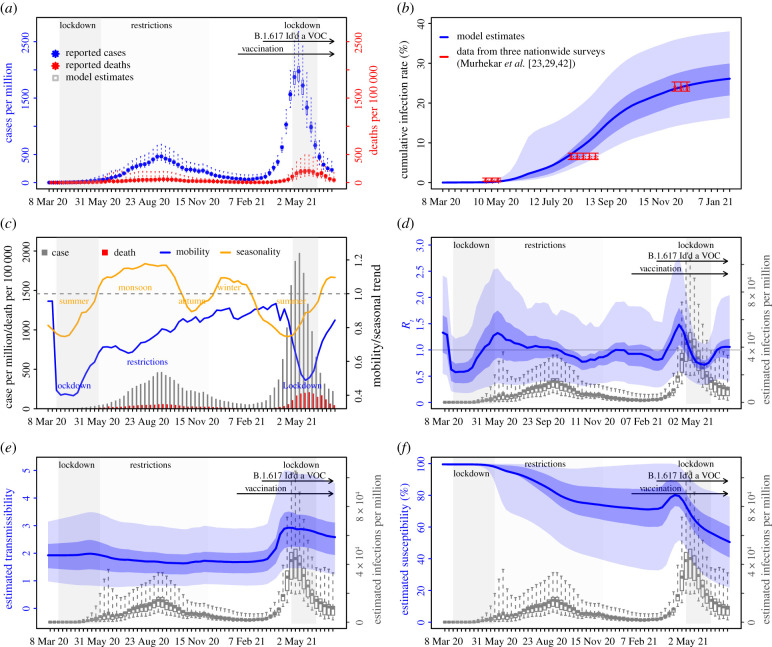
Model-inference estimates and validation. (*a*) Model fit. (*b*) Model validation. (*c*) Observed relative mobility and estimated disease seasonal trend, compared with case and death rates over time. Key model-inference estimates are shown for (*d*) the real-time reproduction number *R_t_*, (*e*) transmissibility *R_TX_* and (*f*) population susceptibility, expressed relative to the population size (i.e. *S_t_*/*N* × 100%). Blue lines and surrounding areas show the estimated mean, 50% (dark) and 95% (light) CrIs. Boxes and whiskers show the estimated mean, 50% and 95% CrIs for weekly cases and deaths in (*a*) and infection rates in (*d*–*f*). Grey shaded areas indicate the timing of national lockdowns (darker) or local restrictions (lighter); horizontal arrows indicate the timing of variant identification and vaccination rollout. In (*c*), for mobility (blue line; y-axis), values below 1 (dashed horizontal line) indicate reductions due to public health interventions. For the disease seasonal trend (orange line; y-axis), values above 1 indicate weather conditions more conducive for transmission than the yearly average and vice versa. Note that the transmissibility estimates have removed the effects of changing population susceptibility, NPIs and disease seasonality; thus, the trends are more stable than the reproduction number (*R_t_* in *d*) and reflect changes in variant-specific properties.

From March 2020 to January 2021, India recorded over 10 million COVID-19 cases (0.77% of its population); however, a nationwide serology survey suggested that approximately 24% of its population had been infected by December 2020 [23]. Accounting for under-detection of infection (electronic supplementary material, figure S1), implemented NPIs, seasonality and vaccination, we used the model-inference system to reconstruct pandemic dynamics in India since March 2020 (figure 1*a*). Model-estimated infection rates closely match with measurements from three nationwide serologic surveys conducted during the early, mid and late phases of the first pandemic wave (figure 1*b*). Our analysis indicates that the two-month-long national lockdown (24 March–31 May 2020) and the less favourable weather conditions during pre-monsoon season (i.e. March–May) probably contributed to initial low infection rates. By mid-May 2020, the model-inference system estimates that only 0.43% (95% CrI: 0.19–1.7%) of the population had been infected (versus 0.73% (95% CI: 0.34%, 1.13%) among adults estimated by serosurvey [29]). As the country lifted its lockdown in June 2020 and entered the monsoon season (June–September), when conditions are probably more favourable for transmission (figure 1*c*), the first pandemic wave began. Nevertheless, continued regional restrictions during June–November 2020 and less favourable weather conditions during the autumn (October–November; see mobility and seasonal trends in figure 1*c*) probably mitigated pandemic intensity. The estimated mean of the reproduction number *R_t_* (i.e. average number of secondary infections per primary infection) was above 1 but less than 1.35 from June to mid-September; in addition, *R_t_* dropped below 1 during October–November (figure 1*d*). By the end of January 2021 when case rates reached a minimum following the first wave, the model-inference system estimates that 26.1% (95% CrI: 19.9–33.0%) of the population had been infected (figure 1*b*).

### 2.2. The second pandemic wave in India and estimated epidemiological characteristics of Delta

Infections resurged dramatically in late March 2021, largely due to the rise of the Delta variant. Despite a week-long second national lockdown implemented beginning 20 April 2021, India reported another 19 million cases during late March–June 2021, about twice the number reported during the previous 12 months. Accounting for under-detection (electronic supplementary material, figure S1), we estimate that 32.3% (95% CrI: 22.4–46.5%) of the population were infected during this three-month period, including reinfections. This intense transmission was probably facilitated by the higher transmissibility and immune evasive capabilities of the Delta variant. Estimated transmissibility increased substantially during the second pandemic wave (figure 1*e*). In addition, estimated population susceptibility increased at the start of the second pandemic wave (figure 1*f*), suggesting the loss of population immunity against Delta. Due to this immune escape, an estimated 50.5% (95% CrI: 21.8–79.0%) of the population remained susceptible at the end of June 2021, despite two large pandemic waves and rollout of mass vaccination (of note, 19% of the population had received at least first dose of vaccine by the end of June 2021). These findings along with the seasonal trends described above suggest that the decline of the second wave was largely due to the NPIs implemented and less favourable weather conditions during March–May, rather than high population immunity.

Combining the model-inference estimates during the first and second pandemic waves in India, we estimated that Delta was able to escape immunity among 34.6% (95% CI: 0–64.2%) of individuals with prior wild-type infection and was 57.0% (95% CI: 37.9–75.6%) more transmissible than wild-type SARS-CoV-2. Estimates are similar under different VE settings (electronic supplementary material, figure S2).

### 2.3. Impact of vaccination and prior non-Delta infection on boosting vaccine-induced immunity

**Figure 2.**
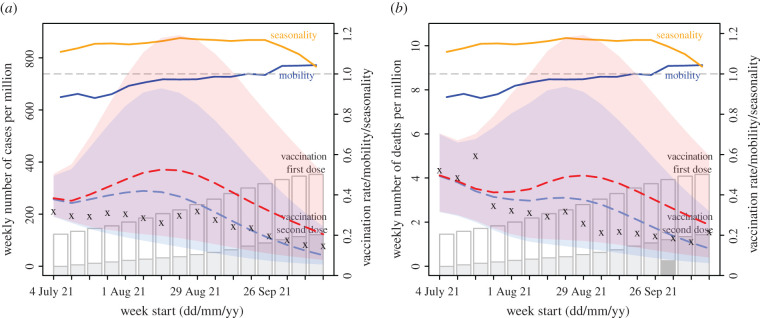
Impact of vaccination. Model projections of weekly number of reported cases (*a*) and reported deaths (*b*) for India during July–mid-October 2021, compared with reported data. Crosses (x) show reported data (left *y*-axis). Red dashed lines show median counterfactual model projections assuming no further vaccination uptake during the 16-week period. Blue dashed lines show median model projections using reported vaccination rates and assuming 90%/95% VE for individuals with prior non-Delta infection after the first/second vaccine dose. Shaded areas with the same colour show projected interquartile ranges. For comparison, estimated seasonality (orange lines), reported mobility (dark-blue lines) and cumulative vaccination uptake (full bar for first dose and filled section for second dose) are overlaid (see right *y*-axis scale). All numbers are scaled per one million people.

**Figure 3.**
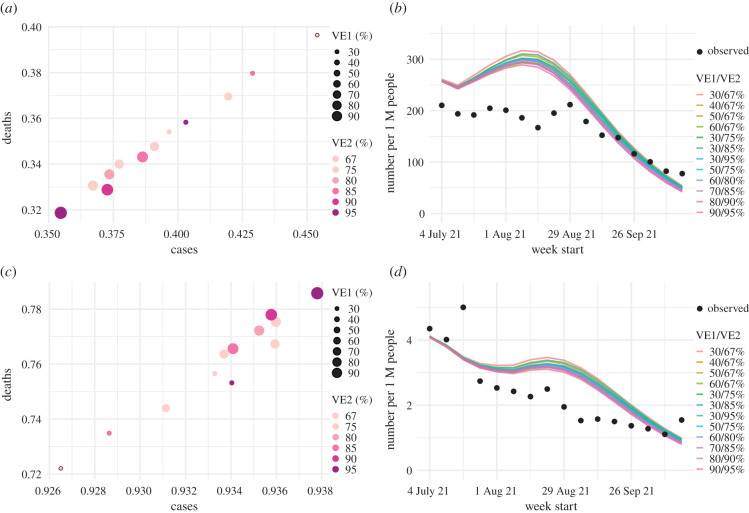
Impact of prior non-Delta infection on immune boosting. Model projections under different VE settings are used to examine the most plausible VE for individuals with prior non-Delta infection, based on projection accuracy: (*a*) the RRMSE and (*c*) correlation between the projected and observed values for cases and deaths, respectively. The size of the dots represents VE for recoverees after the first vaccine dose and the colour represents VE after the second vaccine dose. The dots with the black circles represent the baseline VE setting (i.e. 30%/67% for the first/second dose). For comparison, projected weekly numbers of reported cases (*b*) and reported deaths (*d*) under different VE settings are plotted along with the weekly actuals. For clarity, only median projections are shown here; see example projections including interquartile ranges in figure 2.

Despite the likely conducive conditions during the monsoon season (June–September), easing of NPIs, and relatively high susceptibility estimated at the end of June 2021, cases and deaths in India remained at relatively low levels during July–October 2021. Counterfactual modelling suggests that the faster rollout of vaccination during this period substantially mitigated the epidemic risk (figure 2). Projected cases and deaths assuming no further vaccination uptake are much higher than observed; in contrast, models including the reported vaccination rates more closely match reported cases and deaths (figure 2). Further, models assuming higher VE for non-Delta infection recoverees generated more accurate projections than those assuming no boosting effect (figure 3). The boosting effect appears to be more pronounced for the first vaccine dose (see e.g. figure 3*a* where larger dots, representing higher VE after the first dose, had smaller errors). Overall, the model assuming 90%/95% VE for the first/second dose of vaccine for non-Delta infection recoverees generated the most accurate projections. These projections estimate that vaccination rollout combined with the boosting effect averted 57% of infections during July–mid-October 2021.

## 3. Discussion

Combining epidemiological, behavioural and weather observational data with a comprehensive model-inference system, we estimate the Delta SARS-CoV-2 variant escaped immunity in roughly one-third of individuals with wild-type infection during the previous year and was around 60% more infectious than wild-type SARS-CoV-2. In addition, our analysis suggests the large increase in population receiving their first vaccine dose (approx. 50% by end of October 2021) combined with the boosted VE for non-Delta infection recoverees probably helped mitigate the epidemic intensity in India during July–October 2021.

Previously, we have estimated the changes in transmissibility and immune escape potential for three other major SARS-CoV-2 VOCs: namely, a 46.6% (95% CI: 32.3–54.6%) increase in transmissibility but nominal immune escape for Alpha (i.e. B.1.1.7), a 32.4% (95% CI: 14.6–48.0%) increase in transmissibility and 61.3% (95% CI: 42.6–85.8%) immune escape for Beta (i.e. B.1.351), and a 43.3% (95% CI: 30.3–65.3%) increase in transmissibility and 52.5% (95% CI: 0–75.8%) immune escape for Gamma (i.e. P.1). Compared with Alpha, data from the UK have shown that the secondary attack rate for contacts of cases with Delta was around 1.5 times higher than Alpha (12.4% versus 8.2%), during 29 March–11 May 2021 [2]. In a partially immunized population, the secondary attack rate reflects the combined outcome of the transmissibility of the etiologic agent and population susceptibility to that agent. Consistent with the UK data, our estimates of the relative transmissibility and immune escape potential combine to a 44.1% (95% CI: 4.2–86.6%) higher secondary attack rate by Delta than Alpha (i.e. (1 + 57%)/(1 + 46.6%) × (1 + 34.6%) − 1 = 44.1% increase). This higher competitiveness of Delta over Alpha explains the rapid variant displacement observed in regions previously dominated by Alpha (e.g. the UK and the USA).

In addition, we estimate that 34.6% (95% CI: 0–64.2%) of individuals with acquired immunity from wild-type infection would be susceptible to Delta due to immune escape. This estimate is also in line with Dhar *et al*. [22] reporting a 27.5% reinfection rate during the Delta pandemic wave in Delhi, India, based on a small subset of people with repeated serology measures. In addition to immune escape from wild-type infection, studies have also reported reduced ability of sera from Beta- and Gamma-infection recoverees to neutralize Delta [8,30], suggesting Delta can also escape immunity conferred by those two VOCs. Such immune escape ability would also allow Delta to rapidly replace Beta and Gamma in regions previously hard-hit by those two VOCs, as has been observed in many countries in Africa and South America [5]. More fundamentally, these findings highlight the complex, nonlinear immune landscape of SARS-CoV-2 and the importance to monitor the immune escape potential of new variants against both previous and concurrent circulating variants.

Despite the successful development of multiple vaccines, shortage of supplies—particularly in resource-limited countries—remains an impediment to global mass vaccination [31]. In response, researchers have proposed dose sparing strategies such as fractionation [32] and one-dose vaccination for recoverees [33]. The latter one-dose strategy draws on laboratory studies showing higher vaccine-induced immune response among recovered vaccinees than naive vaccinees (i.e. boosting of pre-existing immunity) [25–28]. Here, we used model-inference estimates and vaccination data in India to test the impact of boosting at the population level. The findings further support the effectiveness of first-dose vaccination for recoverees. In the light of continued vaccine shortages, prioritizing first-dose vaccination thus may be an effective strategy for mitigating COVID-19 burden in countries with high underlying SARS-CoV-2 infection rates.

Due to a lack of detailed epidemiological data (e.g. age-specific and subnational) and thus model simplification, our estimates have uncertainties as indicated by the large credible intervals. Nevertheless, these estimates are in line with independent data from three nationwide serology surveys conducted at three time points during the first pandemic wave in India (figure 1*b*), as well as Delta-related epidemiological data from the UK [2] and Delhi, India [22], as discussed above; these consistencies support the accuracy of our estimates. Unlike estimates from the contact tracing data, however, here we are able to separately quantify the changes in transmissibility and immune escape potential of the Delta variant. In addition, our analysis also suggests high VE of one-dose vaccination among those with prior infection. These findings and the methods used to generate them could support better understanding of future SARS-CoV-2 variant dynamics given local prior infection rates, variant prevalence and vaccination coverage.

## 4. Methods

### 4.1. Data sources and processing

We used reported COVID-19 case and mortality data to capture transmission dynamics, weather data to estimate infection seasonality, mobility data to represent concurrent NPIs and vaccination data to account for changes in population susceptibility due to vaccination in the model-inference system. COVID-19 case and mortality data from the week of 8 March 2020 (the first week COVID-19 deaths were reported in India) to the week of 17 October 2021 came from the COVID-19 Data Repository of the Center for Systems Science and Engineering at Johns Hopkins University [34,35]. Surface station temperature and humidity data were accessed using the ‘rnoaa’ R package [36]. We then aggregated these data for all weather stations in India (*n* = 390 stations) with measurements from January 2020 to October 2021 and calculated the average for each week of the year. Mobility data were derived from Google Community Mobility Reports [37]; we aggregated all business-related categories (i.e. retail and recreational, transit stations and workplaces) in all locations in India to weekly intervals. Vaccination data (first and second dose) were obtained from Our World in Data [38,39].

### 4.2. Model-inference system

The model-inference system was developed and described in detail in our previous study [24]. Below we describe each component in brief.

#### 4.2.1. Epidemic model

The epidemic model follows an SEIRSV (susceptible-exposed-infectious-recovered-susceptible-vaccination) construct per equation (4.1)

4.1 $$\begin{array}{rl} \frac{dS}{dt}= & \frac{R}{L_{t}}-\frac{b_{t}e_{t}m_{t}\beta_{t}IS}{N}-\varepsilon -v_{1,t}-v_{2,t},\\ \frac{dE}{dt}= & \frac{b_{t}e_{t}m_{t}\beta_{t}IS}{N}-\frac{E}{Z_{t}}+\varepsilon ,\\ \frac{dI}{dt}= & \frac{E}{Z_{t}}-\frac{I}{D_{t}}\\ \mathrm{and}\;\;\frac{dR}{dt}= & \frac{I}{D_{t}}-\frac{R}{L_{t}}+v_{1,t}+v_{2,t}, \end{array}\}$$

where *S*, *E*, *I*, *R* are the number of susceptible, exposed (but not yet infectious), infectious and recovered/immune/deceased individuals, *N* is the population size and *ε* is the number of travel-imported infections (here nominally set to 1 per 10 days per 1 million people). The model is run stochastically and includes the following key components:

(i) Virus-specific properties, including the time-varying variant-specific transmission rate $\beta_{t}$, latency period *Z_t_*, infectious period *D_t_* and immunity period *L_t_*. Note the subscript, *t*, denotes time in weeks, as all parameters are estimated for each week as described below.
(ii) The impact of NPIs. Specifically, we use relative population mobility (see data above) to adjust the transmission rate via the term *m_t_*. To further account for potential changes in effectiveness, the model additionally includes a parameter, *e_t_*, to scale NPI effectiveness.
(iii) The impact of vaccination, via the terms *v_*1*,t_* and *v_*2*,t_*. Specifically, *v_*1*,t_* is the number of individuals successfully immunized after the first dose of vaccine and is computed using vaccination data and vaccine efficacy for first dose; and *v_*2*,t_* is the additional number of individuals successfully immunized after the second vaccine dose (i.e. excluding those successfully immunized after the first dose).
(iv) Infection seasonality, computed using temperature and specific humidity data as described previously (see supplemental material of Yang & Shaman [24]). The estimated relative seasonal trend, *b_t_*, is used to adjust the relative transmission rate at time *t*.

#### 4.2.2. Observation model to account for under-detection and delay

Using the model-simulated number of infections occurring each day, we further computed the number of cases and deaths each week to match with the observations, as done in Yang *et al*. [40]. For example, for case data, we include (i) a time-lag from infectiousness to detection (i.e. an infection being diagnosed as a case) to account for delays in detection and (ii) an infection-detection rate (*r_t_*), i.e. the fraction of infections (including subclinical or asymptomatic infections) reported as cases, to account for under-detection. Specifically, to compute the model-simulated number of new cases per week, we used the following observation model:

4.2 $$C_{t}=\overset{k=7t+7}{\underset{k=7t+1}{\sum }}\overset{s=S}{\underset{s=1}{\sum }}p_{s}r_{t}I_{k-s},$$

where *C_t_* is the model-simulated number of new cases during week *t*. In the inner summation, *I_k−s_* is the model-simulated number of new infectious individuals from *s* days before their detection as cases; *s* is the time from infectiousness to detection (up to a maximum delay of *S* days; *S* was set to 14 days here) and *p_s_* is its probability distribution (here modelled as a gamma distribution with mean *T_mean_* and standard deviation *T_sd_*). That is, the inner summation computes the model-simulated number of cases per day, given the infection-detection rate (*r_t_*) and time-lag in detection; the outer summation computes the weekly total by aggregating daily estimates over the 7 days of the week. Note that *T*_mean_, *T_sd_* and *r_t_* are estimated using the model-inference system along with other model parameters and state variables and the weekly totals are used for model-inference, fitting to the observed data (see below).

#### 4.2.3. Model-inference and parameter estimation

The inference system uses the ensemble adjustment Kalman filter (EAKF) [41], a Bayesian statistical method, to estimate model state variables (i.e. *S*, *E*, *I*, *R* from equation (4.1); electronic supplementary material, figure S3) and parameters (i.e. $\beta_{t}$, *Z_t_*, *D_t_*, *L_t_*, *e_t_*, from equation (4.1) as well as *r_t_* and other parameters from the observation model; electronic supplementary material, figure S4). Briefly, the EAKF uses an ensemble of model realizations (*n* = 500 here), each with initial parameters and variables randomly drawn from a prior range (see electronic supplementary material, table S1). After model initialization, the system integrates the model ensemble forward in time for a week (per equation (4.1)) to compute the prior distribution for each model state variable and parameter, as well as the model-simulated number of cases and deaths for that week. The EAKF then combines the prior estimates with the observed case and death data for the same week to compute the posterior per Bayes' theorem [41]. Importantly, the EAKF adjusts the model state variables and parameters following each assimilation of observations, instead of working from a fixed set of parameter proposals. As such, the continuous parameter adjustment allows for time-evolving estimation of these values.

#### 4.2.4. Estimating the immune escape potential and changes in transmissibility for Delta

To identify the most plausible combination of changes in transmissibility and level of immune evasion, per methods developed in [24], we ran the model-inference, repeatedly and in turn, to test 14 major combinations of these two quantities and select the best-performing run. Based on the best-performing model estimates, we then computed the variant-specific transmissibility (*R_TX_*) as the product of the variant-specific transmission rate ($\beta_{t}$) and infectious period (*D_t_*). To reduce uncertainty, we averaged transmissibility estimates over the first pandemic wave and the period when Delta is dominant, separately. We then computed the average change in transmissibility due to Delta as the ratio of the two averaged estimates (i.e. after : before the rise of Delta). To quantify immune evasion, we recorded the changes in immunity at each time step *t* as *ΔImm_t_* = *S_t_*_+1_ − *S_t_* + *i_t_* (with *S_t_* as the susceptibility at time *t* and *i_t_* as the new infections occurring at time *t*); we then sum over all *ΔImm_t_* estimates during the second wave when the new variant is predominant to compute the total change in immunity due to the new variant. We further compute the level of immune evasion as the ratio of the total change in immunity during the second wave to the model-estimated population immunity at the end of the first wave (i.e. the baseline before the new variant surge). This ratio provides an estimate of the fraction of individuals previously infected who are susceptible to re-infection with the new variant.

Model-inference was done continuously from the week starting 8 March 2020 to the week starting 27 June 2021. To account for model stochasticity, we repeated the model-inference process 300 times, each with 500 model realizations, and summarized the results from all 150 000 model estimates. As a sensitivity test and part of the effort to examine the impact of prior non-Delta infection on VE, we performed the analysis using 12 different VE settings (see details below).

### 4.3. Model validation using independent data

To compare model estimates with independent observations not assimilated into the model-inference system, we identified three measurements of cumulative infection rates from three nationwide serology surveys in India: (i) the first national serosurvey conducted during 11 May–4 June 2020 (*n* = 28 000 adults 18 years or older); [29] (ii) the second national serosurvey conducted during 18 August–20 September 2020 (*n* = 29 082 individuals 10 years or older); [42] and (iii) the third national serosurvey conducted during 18 December 2020–6 January 2021 (*n* = 28 598 individuals 10 years or older). [23] To account for the delay in antibody generation, we shifted the timing of each serosurvey 14 days when comparing survey results to model-inference system estimates of cumulative infection rates in figure 1*b*.

### 4.4. Estimating the impact of vaccination and prior non-Delta infection on boosting vaccine-induced immunity

We generated retrospective projections of cases and deaths from the week starting 4 July 2021 to the week starting 17 October 2021 (i.e. 16 weeks following the model-inference period), under various vaccination and VE settings. We considered four levels of VE for those recovered from non-Delta infection: (i) no boosting effect, i.e. using the same VE values as those without prior infection, (here, we set VE at 14 days after the first dose (VE1) to 30% and at 7 days after the second dose (VE2) to 67%, based on data for the AstraZeneca vaccine against Delta); [15] (ii) higher VE for the first dose but no future boosting for the second dose (here, VE1 is set to 40%, 50% or 60%, and VE2 fixed at 67%); (iii) higher VE for the second dose but not first dose (here, VE1 is fixed at 30% and VE2 set to 75%, 85% or 95%); and (iv) higher VE for both doses (here, VE1/VE2 are set to 50%/75%, 60%/80%, 70%/85%, 80%/90% or 90%/95%). To test the impact of vaccination, in addition to projections using reported vaccination rates, we also generated counterfactual projections assuming no further vaccination during the 16-week period.

For all projections, the model was initiated using model-inference estimates made at the week of 27 June 2021, except for the infection-fatality risk (IFR). For IFR, estimates were decreasing during June 2020 (electronic supplementary material, figure S1B) and model-inference extended to the end of July 2021 showed continued decreases, probably due to improved healthcare and increased protection from prior infection or vaccination. We thus assumed that IFR would decrease linearly for the first six weeks of the projection period and then flatten and remain at that low IFR until the week of 17 October 2021. To account for NPIs, we used mobility data during the week of 4 July 2021 to the week of 17 October 2021. As for the model-inference runs, we repeated the projections for each scenario 300 times (each with 500 model realizations) and summarized the projections from all runs. To evaluate the projection accuracy, we computed the relative root-mean-square-error (RRMSE) and correlation between the projected and observed values for cases and deaths, respectively.

## Data Availability

All data used in this study are publicly available as described in the ‘Data sources and processing’ section. All source code and data necessary for the replication of our results and figures are made publicly available at https://github.com/wan-yang/covid_voc_delta. Electronic supplementary material is available online [43].
